# Steroid-induced mania in a patient with previously well-controlled organic bipolar 1-like affective disorder secondary to acquired brain injury: case report and literature review

**DOI:** 10.1007/s44192-024-00061-w

**Published:** 2024-03-08

**Authors:** Jacob D. King, Thomas Elliott, Alexandra Pitman

**Affiliations:** 1 Division of Psychiatry, Imperial College London, London, UK; 2 Central and North West London NHS Foundation Trust, London, UK; 3 Camden and Islington NHS Foundation Trust, London, UK; 4 Division of Psychiatry, University College London, London, UK; 5 East London Foundation Trust, London, UK

**Keywords:** Manic episode, Psychoses, substance-induced, Brain injuries, Bipolar disorder, Neuropsychiatry

## Abstract

Steroid-induced neuropsychiatric sequelae are common, and pose significant risks to people usually receiving glucocorticoids in the context of physical illness. Steroid-induced mania and hypomania are the most common of the acute complications, yet despite great progress in understandings in neurophysiology there are no recent studies which review the factors which might predict who will experience this severe complication, nor are there consensus guidelines on management. We report the unusual case of a woman in her 50s admitted to a psychiatric unit with steroid-induced mania despite compliance with two mood stabilisers, several days after the administration of a Dexamethasone and Docetaxel chemotherapy regime adjunctive to lumpectomy for breast cancer. She had previously been diagnosed with an organic affective disorder (with classical bipolar 1 pattern) following severe ventriculitis related to ventricular drain insertion for obstructive hydrocephalus secondary to a colloid cyst. She had no psychiatric illness before this brain injury, but has a maternal history of idiopathic bipolar 1 affective disorder. Her episode of steroid-induced mania resolved following use of sedative medications, continuation of her existing mood stabilisers, and reductions of the steroid dosing in collaboration with her oncology team, which also protected her from further manic relapses during continued chemotherapy. Established mental illness, a family history, and acquired brain injury may reflect risk factors for steroid-induced mania through currently unclear pathways. Future epidemiological studies could better confirm these observations, and basic neuroscience may look to further explore the role of extrinsic glucocorticoids in the pathophysiology of affective disorders.

## Introduction

Steroid-induced mania (SIM) and steroid-induced hypomania are the most common acute neuropsychiatric complications of high-dose glucocorticoid therapy [1]. However, mania induced by extrinsic glucocorticoids in patients with a predisposition to manic episodes, principally those with a preexisting diagnosis of bipolar affective disorder (BPAD), is uncommon [2]. Whilst in part this might represent the prophylactic benefit of concurrent mood-stabilising medication [3, 4] little is known about the role of extrinsic glucocorticoids in the pathophysiology of mania. In this article we present an unusual case of steroid-induced mania on a background of an organic affective disorder with classical bipolar-1 pattern in remission, with a heritable component, which provides a novel opportunity to contextualise the literature on the use of high dose glucocorticoids in patients with previous manic episodes. This article was written in keeping with CARE guidelines.^1^

## Case report

‘M’, a woman in her late 50s, was brought to the emergency department (ED) two days following her second cycle of fortnightly chemotherapy adjunctive to lumpectomy for stage 2 breast cancer. This had consisted of intravenous Dexamethasone 16 mg per day over 3 days (106.7 mg prednisolone equivalent daily), in preparation for Docetaxel on the third day. On assessment by the duty psychiatrist M was highly aroused, irritable, pacing around the department, laughing and muttering to herself. Her mood was objectively elated with an expansive, labile affect. Her speech was pressured, with a loosening of topical associations. She was easily distracted, and incoherent for parts of the conversation, often returning to erotomanic beliefs about famous musicians. She appeared to be responding to auditory hallucinations but did not confirm this when asked. At the time her Young Mania Rating Scale (YMRS) score was 26/60 (a score of 25 best represents the threshold for severe mania) [5].

M had been diagnosed with an organic bipolar 1-like affective disorder by a tertiary neuropsychiatry department 10 years previously and was most recently prescribed Lithium carbonate 800 mg and Lamotrigine 225 mg both once nightly, with her family and mental health support worker reporting good compliance. Lithium levels routinely pre-chemotherapy and on presentation to the ED were both within the therapeutic range. She took no other routine medications, but had been given a seven day post-chemotherapy regime of Domperidone and Aprepitant for symptomatic relief of nausea, and Fluconazole as a prophylactic against fungal infection. M had already completed a Cyclophosphamide and Doxorubicin regime 4 months earlier without significant side effects, and reported having been naïve to corticosteroids until this point.

Physical observations were normal and blood markers of infection, electrolytes, and liver, renal and thyroid profiles returned within normal parameters. Her lithium levels were within therapeutic range at 0.79mmol/L. She declined a urine drug screen at the point of admission but later denied any history of drug use for some years, reporting only "speed and blues" in her 20s, which was corroborated by her sister. Of significance, M’s mother has a diagnosis of idiopathic BPAD, and had been admitted with a manic episode herself contemporaneously at a different psychiatric unit while M was under our care. There were also significant childhood stressors in M’s earlier years related to her mother’s poor mental health, and M had been intermittently cared for by other family and community members. M did well at school, meeting all academic and social expectations, subsequently worked full-time in a semi-skilled profession and maintained long term relationships. Until her presentation with post-chemotherapy mania M had 3 years of stability on her current medication, and was being seen regularly by her sister and community mental health Intensive Support Team worker three times per week.

M’s only past medical history was of severe brain injury 13 years earlier. Having presented to the ED with reduced consciousness, vomiting, and later entering status epilepticus following a year of chronic headaches, computerised tomography identified marked obstructing hydrocephalus secondary to a 10 mm colloid cyst at the confluence of the right lateral ventricle and foramen of Monro. Bilateral ventricular drains were inserted, and several days later the tumour was successfully removed. However, the procedure was complicated with ventriculitis caused by a Vancomycin-resistant enterococcus. She improved slowly over months of neuro-intensivist treatment, and following a year of neurorehabilitation M returned to work part-time. Over the following months the family noted increasing difficulty with M’s attendance to personal care. Although she had not previously had any psychiatric history M became socially withdrawn with poor motivation, anhedonia, irritability, insomnia, fatigue, impairment of executive function, and subjective memory deficit, although mood disturbance was not evident at this time. She required psychiatric admission, and assessment by a tertiary neuropsychiatric unit formulated a diagnosis of organic depressive mood disorder secondary to brain injury. Her symptoms did not respond to two different selective serotonin re-uptake inhibitors, nor Mirtazapine.

Months later, M presented to ED agitated and not having slept for days, with paranoid ideas concerning neighbours and erotomanic beliefs about a famous musician. Her house was in disarray, smelling of urine with rubbish piled up in her bed and neighbours reporting music having been played at a very high volume throughout days and nights. She was admitted to a different psychiatric unit for several months. Further review by a second tertiary neuropsychiatric unit revised her diagnosis to organic bipolar mood disorder, an Olanzapine/Fluoxetine regime was advised and some stability was initially achieved.

Over the following years M’s daily functioning progressively declined and she moved into supported accommodation. Despite trials of other atypical antipsychotics, nimodipine, and several combinations of mood stabiliser dual therapy, her symptoms proved difficult to treat and resulted in either depressed or manic relapses leading to involuntary hospitalisation a further four times (two of which lasted over a year). Usually it was unclear what the precipitant was for these presentations, although her sister and clinicians considered stressful life events like her mother becoming unwell and her household heating breaking, as well as on at least one occasion medication non-compliance contributing; M had been sceptical of the benefits of medication albeit largely compliant (and always closely monitored) for a number of years after her mental health difficulties began. When depressed she presented with poor self-care, lying in bed all day, unable to wash, cook, shop or clean, and when manic, she presented with prolific spending, overfamiliarity, impulsivity, poor sleep, paranoia regarding neighbours and medication, and (less frequently) complex repetitive behaviours. The latter included spelling out variations on “floral war dance huh huh huh” in different written mediums (broken biscuits, powered sugar, cigarette butts), reinstating these words if cleared away by hospital staff. During one admission with a depressive presentation eight treatments with electro-convulsive therapy was followed by partial remission from symptoms. Throughout her illness M displayed hoarding behaviours and slowly progressive multi-domain cognitive impairment. Serial neurocognitive assessments demonstrated slow decline, principally in recall and recognition memory, lexical fluency, set-shifting and overall executive function, implicating anterior frontal and bi-temporal areas. Magnetic resonance neuroimaging at 1, 9 and 11 years post-event noted a moderate degree of generalised cerebral atrophy, and a persistent increase of T2 signal intensity of the anterior frontal lobes bilaterally, most significant on the right superiorly, and in keeping with post-operative changes. There was no evidence of another neurodegenerative process.

During the current presentation M was admitted to our female inpatient psychiatric ward under section 2 of the United Kingdom’s Mental Health Act 1983, and treated with Clonazepam 2 mg QDS and 25 mg QDS of Promethazine in addition to the continuation of her regular mood-stabilising medications Lithium and Lamotrigine. M made a marked recovery, with a YMRS score of 0 at day 7. On discussion with her oncology team it was decided that her next Dexamethasone regime would be reduced to a single 16 mg IV dose on the same day as Docetaxel administration. We recommended Clonazepam 2 mg QDS for subsequent chemotherapy sessions, and continued this for two days following her treatment, followed by a weaning regime over the subsequent 7 days. After recovery from this manic episode she scored 73/100 on Addenbrookes’ Cognitive Examination (ACE)-III, consistent with previous assessments. She was then discharged home with a plan for the same benzodiazepine cover regime for all remaining cycles, to be supervised by her oncology team, and for increased social support from M’s community mental health team care coordinator. Six months later M remained stable in her mental state, and was able to complete this adjusted chemotherapy regime without further psychiatric complication.

## Discussion

In contextualising M’s case we reviewed the literature available on the topic in searches in EMBASE, MEDLINE and PsychINFO via Ovid, and grey literature in Google Scholar.

Manic symptoms that do not present until later life are more likely to reflect an acquired aetiology [6]. Causes of secondary manic episodes include seizure, neoplasm, inflammation, infection, metabolic disturbances, acute brain injury and neurovascular events [7, 8]. Several reviews have identified that a family history of affective disorder and right hemispheric lesions may increase risk of a secondary manic episode following traumatic brain injury (TBI) [8, 9]. The sequelae of many primary neurological, endocrine and rheumatological disorders are also implicated, as are a range of extrinsic agents particularly drugs with dopaminergic and serotonergic effects as well as corticosteroids, thyroxine, among other reports of idiosyncratic reactions [10–13]. Yet the production of a persistent organic affective disorder (ICD-10 code F06.3) with a psychiatric profile analogous to idiopathic bipolar 1 affective disorder, characterised by depressed, manic or mixed states, is much less common than isolated secondary manic episodes [14]. Acquired brain injuries appear nearly unique in this regard, albeit a handful of cases including frontal lobe malignancy [15], and stroke [14] are also described. From small samples Jorge et al. [16] suggest 6.5% to 9% of those who sustain a TBI develop a BPAD like illness, with a relative risk between 1.1 and 5.3 compared to people without TBI in their past [17]. These authors highlight that short follow-up periods and effective treatment limit what clinically can be said about these cases of TBI related BPAD-like illnesses, and whether these cases in fact represent an acute post-TBI manic episode followed by depressive illness without any future manic states, or indeed a persistent organic affective disorder with BPAD-1 like pattern. A clinical distinction between two psychopathological processes in secondary mania and acquired BPAD-1 like illnesses must therefore be made. Importantly however, there do not appear to be any previously reported cases of the combination of the two: secondary mania occurring in an individual with an organic bipolar 1-like affective disorder.

Steroid-induced mania is a common culprit of secondary mania [14]. Steroid-induced acute neuropsychiatric sequelae, which include steroid-induced mania, are common, and are reported across glucocorticoid preparations and doses [18–21]; estimates suggest around 5–10% of patients administered high-dose preparations experience significant neuropsychiatric sequelae [22, 23]. Authors remind us that historically, “diverse affective, behavioral, and cognitive syndromes [following extrinsic steroid administration] were lumped together under the term *steroid psychosis*”, [24] and throughout this article we have therefore been sensitive to search literature written using synonymous terms. Some authors anticipated that the coronavirus pandemic had the potential to increase numbers of cases of steroid-induced neuropsychiatric sequelae given that Dexamethasone treatment was a mainstay of the inpatient treatment of severe COVID-19 [25]. However, this did not appear to materialise in reports; a meta-analysis of first-episode mania following COVID-19 identified only 23 reported cases, of which fewer than half (47.8%) had received steroids [26]. That review however excluded individuals with preestablished BPAD. Despite the familiarity of clinicians with steroid-induced psychiatric sequelae, there continues to be a paucity of documented cases, and we have been unable to identify any study yet to systematically identify risk factors.

M’s case is typical of other reports of steroid-induced mania: occurring at higher rates in women, and characterised by irritability, poor sleep, mood lability, suspected auditory hallucination, and which resolve quickly following the removal of steroids. Her Naranjo Adverse Drug Reaction Probability Scale [27] score was 7 indicating probable adverse drug event. This reflected the timing of a response clinically in keeping with previously described cases of steroid-induced mania, which responded quickly when the agent was withdrawn, did not re-occur at lower doses with appropriate treatment, and was without any other suspected drug-induced cause. Incidence of SIM among patients taking steroids is thought to be directly related to dose but not necessarily length of course [1]. A bimodal pattern appears to operate, with some individuals experiencing onset of symptoms 3–4 days from initial dose and others, as in the case of M, from 11 days after initial treatment [21]. While the United Kingdom’s National Institute of Health and Care Excellence (NICE) guidelines on the use of oral corticosteroids advise caution in patients with “psychoses or severe affective disorders”, it does not advise total contraindication, and no evidence is cited as to likely mechanisms [28].

Previous reports from very small numbers of patients had revealed a signal that mood stabilising medications may offer prophylactic benefit against steroid-induced mania [3, 4]. However the present case, in which mania was induced despite compliance with two mood stabilising medications at doses demonstrated to reduce the incidence of mania in patients with BPAD, might imply either an alternative causal mechanism for mania circumventing the established therapeutic effects of these medications, and/or that the other risk factors in this patient’s case (family history, acquired brain injury) superseded prophylactic benefit.

Increasing evidence for a range of predisposing factors to developing symptomatology consistent with BPAD has developed over the last 5–10 years, with good evidence for a high degree of heritability [29] and a notable implication of the hypothalamic-pituitary adrenal (HPA) axis. Extrinsic glucocorticoids have a complex interaction with the human HPA axis [30]. However the exact physiological mechanism by which extrinsic glucocorticoids induce mania remains unclear. Recent evidence links exposure to extrinsic steroids to aberrations of white matter integrity and volumetric grey matter differences [31] notably of the caudate which has frequently been implicated in the neuroanatomical profiles of BPAD [32]. There also appears to be a potential role of HPA dysfunction in modulating the experience of traumatic life events [33], particularly in those with parental history of BPAD [34], wherein a ‘hyperactive’ HPA, might mediate the effects of childhood adversity and dysregulated parenting style on risk of mood disorder, superimposed on a particular genetic risk profile, expressed phenotypically as symptoms of BPAD [35]. While good evidence now demonstrates the interconnectedness of the HPA with other neurotransmitter systems [36], and clinical trials demonstrate the antidepressant effect of agents targeting neurosteroid pathways [37, 38], there is limited evidence linking this understanding with human HPA function following extrinsic glucocorticoid administration. Nor is there evidence describing the role of potential confounders, notably recreational drug use, sleep disturbance [39] or brain injury.

Without clear physiological causal mechanisms there are several clinical aspects of this relationship which remain unclear. First, whether glucocorticoid medications lead to mania directly, or rather “unmask” a predisposition of potentially genetic, or acquired origin [40]. We have been able to identify descriptions of only six other clinical cases of SIM in individuals with pre-existing idiopathic BPAD [41–46], only one discusses a potential physiological cause, highlighting the potential mediating role of poor sleep [43]. It remains only clinical suspicion that severe mental illness predisposes to SIM. Furthermore we can find only one other description of steroid-induced mania in a patient with an acquired brain injury (albeit without resulting organic BPAD like illness as in our case) and without preceding psychiatric illness, who also had frontal lobe injury in this case following stroke [47]. Following on, it remains unclear whether one episode of SIM heralds the later diagnosis of a severe mental illness as has been shown to be the case for other ‘first episode’ drug-induced psychoses [11, 48]. Older review articles suggest that previous psychiatric sequelae of steroids do not necessarily predict future recurrence [21]. However, an analysis of nationwide British general practice data reported a hazard ratio of 1.82 for likely SIM among those with a previously documented episode of mania, compared to matched unexposed persons [49]. Unfortunately the causes of previous manic episodes were not reported in this study, perhaps due to the use of routine data, and therefore it is unclear whether prior mania was related to previous steroid use or indeed related to another cause, including idiopathic BPAD. In a small case series of 9 previously well Japanese patients who developed SIM, several went on to experience manic episodes after further steroid therapy, with authors suggesting that this may represent an acquired predisposition to SIM following initial extrinsic steroid use [50].

We propose that our current case fits a conceptual model of SIM in the context of both an acquired and hereditary predisposition to mania, together perhaps representing a risk profile significant enough to supersede the presumably protective effects of two mood stabilising medications.

Treatment of SIM has been the topic of much interest in the literature, but while no institutional guidelines exist for managing cases of SIM, broad principles can be inferred from the treatment of other drug-induced psychiatric presentations. An alteration of the regime of culpable glucocorticoid medication appears universally advised where clinically possible, whether this means cessation, or reduction in the dosing or frequency of administration. Secondly, a host of case reports and case series individually report on specific pharmacological agents that have resolved their patients’ SIM. In a systematic review of studies describing steroid-induced mental disorders in cancer patients, the pharmacological approaches described indicated that the majority of clinicians opted for mid to long term continuation of a regular anti-psychotic or mood-stabilising medication (most frequently Lithium or Olanzapine), or otherwise short-term cover with sedative medications (usually benzodiazepines or first-generation antihistamines) during continued but adjusted steroid treatment [51]. The findings of this review conclude that most cases of SIM resolve within 7 days of treatment or alteration of the steroid regime. In M’s case, her cancer diagnosis, chemotherapy, and mother’s deterioration requiring admission clearly represented a stressful period in her life. We surmise from this that prior relapses may have been in part precipitated by psychosocial stressors, in the absence of steroids. As with all psychiatric patients, support during periods of psychosocial stress and modification (where possible) of other relapse triggers are important components of care plans.

## Conclusions

This is the first case of which we are aware in which an individual with an acquired cause of a bipolar 1-like affective disorder has experienced steroid-induced mania. It is particularly interesting because of a maternal history of idiopathic BPAD and that the episode of SIM was in the context of compliance with two mood stabilising medications. The case has highlighted the complexities and uncertainties surrounding SIM, and the potential that a better understanding of risk factors for SIM and evidenced treatment approaches could offer affected patients. To date it remains unclear whether genetic or acquired risk factors do confer a vulnerability to steroid-induced mania, which could be explored with large clinical cohorts. Further understanding these issues may also offer translatable benefits to other affective disorders.

## Patient’s perspective

M has read this article and gave informed capacitous written consent to publication. She did not wish to provide any further comments.

## Data Availability

Not applicable.

## Footnotes

1 https://www.care-statement.org/checklist.
